# The Family Physician*Read at May, 1906, meeting of Brazos Valley Mcdical Association, Hearne, Texas

**Published:** 1907-01

**Authors:** D. Monroe

**Affiliations:** Cameron, Texas


For Texas Medical Journal.

### The Family Physician.*

BY D. MONROE, M. D., CAMERON, TEXAS.

The family physician has always occupied a specially peculiar and responsible position in society, with reference to its individual members. For he is both the confessional to the home and counselor to the household, as well as their medical adviser and hygienic guardian, as his advice is always accepted without question and his judgment is implicitly relied upon without criticism.

Within the family circle he has knowledge of all things in particular, and everything in general: from the skeleton in the closet to the pictures in the parlor. Besides, all personal and family secrets are disclosed to him without mental reservation. These are all revealed in perfect confidence and secureness, without hesitation, and so sacred are they that the statutes recognize and acknowledge their sanctity by legalizing this mantle of secrecy.

While the lawyer lives and fattens on the vices and passions of society, and grows opulent off of the contention and strife of its

*Read at May, 1906, meeting of Brazos Valley Medical Association, Hearne, Texas individual members, the physician obtains a bare living for himself and family, by binding up their wounds, ministering to their misfortunes and alleviating their sufferings; and when death is inevitable, he even smooths the path down to the River Styx, by mitigating the throes of death. For enduring fame in his chosen profession can only be won through the altruism—"That the welfare of others must be the dominating principle of his life," without thought of his own convenience, ease or comfort. Therefore, to be a good physician one must bury selfishness, crush ambition and trust to the hereafter for a retributive justice.

Time was but a few years back when the physician was only called upon to heal the sick and cure the afflicted by means of his specific remedies, but within the last decade his relation to society in this particular has very materially changed from the dispenser of his curative remedies to a sanitary guardian and hygienic teacher to those in health, by explaining to them how they may escape the pains and sorrows of ill-health, by pointing out to them the specific causes of their ailments; and so today this is the physician's broadest field of usefulness, both to the community and the country. For, knowledge having supplanted empiricism and quackery being deposed by the science of medicine, the medical student of today devotes more time and attention to the scientific and clinical study of etiology than he does to the treatment of the diseases. So his research in this particular branch of his study has revolutionized the practice of medicine and changed the physician's relation to society, and even his duties thereto, by adding a new and distinct branch to medicine.

### PREVENTIVE MEDICINE.

What greater benificence to humanity can be adduced than that which is evidenced by the scientific student of medicine, who after ardent and patriotic research, discovers the specific cause of disease, and then devises means of immunity? The potentialities of this truth can better be comprehended when we realize that 22 per cent of all deaths in the United States are attributable to these preventable diseases; and even the pestilences, which swept this world in the past, are also placed in the same category, it being an economic fact, that the life of its citizenship is of value to the State in proportion to his productive capacity.

Therefore, every means which tend to increase longevity, by restricting and controlling diseases, is of great pecuniary importance to the State and country. Hence, it is the plain duty of the legislative branch of our government to appropriate all funds necessary to foster and facilitate this great and good work; for the food we eat, the water we drink, and the very air we breathe are all liable to contamination, and thus become inimical to health, consequently, they all require scientific supervision.

Especially should the water supply of the towns and cities be closely scrutinized, as the water oftentimes is responsible for endemic diseases, such as diarrhea, dysentery and even typhoid fever, by being impregnated with decomposed animal matter or decayed vegetable substances. When we have a heavy downpour of rain, following a long dry spell of weather, we may expect all well water to be polluted with such surface washings.

So, if every town would abolish the out-door closet, with its sink, and construct a good system of sewerage with an abundance of flush water, and connect therewith all closets, the said town would soon be free from typhoid fever, while the prevalence of diarrhea and dysentery would be notably lessened.

The recent epidemic of yellow fever in the South Atlantic and Gulf States was controlled and held in abeyance by heeding scientific truths and proven theories—by replacing the shotgun with the mosquito-net—by substituting coal oil for the smudge—by scientifically ferreting out and finding out the true cause of the spread of yellow fever, and then confining all efforts to the elimination of this one palpable cause. Thus, we escaped a scourge of this terrible malady which had heretofore proved so destructive to life and panicky to the living.

Scientific medicine reduced the mortality of yellow fever from 33 per cent to 6 per cent, and thus divested this disease of much of its former terrors. If the physician did no more than to stamp out this one terrible malady and menace to our Southern country, he should receive the plaudits and the gratitude of the whole human race, but this is only one stroke in protecting human life.

But a few years ago we were threatened with a scourge of smallpox which, in years gone by, claimed its victims from all stations in life, excepting and respecting no one. But thanks to Dr. Jenner, we have the key to the situation and locked up, and starved out, this most loathesome of diseases, by rendering the community immune by vaccination.

The day is dawning when our Southern country with its fertile lands and ideal climate, free from the wintry blast of the North and the sultry air of the tropics, will be healthful and free from malaria. This will be an accomplished fact when we educate ourselves and the people to know that a branch of the mosquito family disseminates malarial poison, and by draining their breeding places, we can exterminate the mosquito, and thus be free from malaria—the ban of our Southland.

Within recent years tuberculosis has been catalogued among the infectious diseases by Dr. Koch. Now this disease is known to be transmitted by a specific microbe which is contained in the sputa of the patient. And so, by rendering this sputa innocuous by incineration, we can check the dissemination of this virulent disease, and can reduce the mortality in such cases.

Preventive medicine has raised the average age of man from 29 to 33 years, and the time is at hand when it can be increased to 35 years; which can now even be accomplished with the lights before us, together with the present status of knowledge, if we were only willing to invade the assumed rights and abridge the personal liberties of the few for the benefit of the many. This can and will be done as soon as we are educated sufficiently to understand and appreciate the importance of such a step, to which we are making rapid strides, for the exponent of preventive medicine in Texas wields more authority than the chief executive of the State, because he is autocratic in his power, as his edicts are absolute and his decisions are final, from both of which there are no appeals. He can stop commerce and traffic at a word, and detain the United States mail by a stroke of his pen. He deprives men of their boasted personal liberty and disregards the writ of habeas corpus, and ignores the writ of injunction. All these powers are delegated to him for the sole purpose of protecting the public health. These are the laurels of preventive medicine and these are the achievements of the scientific physician.

Gentlemen of the Brazos Valley Medical Association, we all should not only feel a laudable pride in living in this enlightened age of medical progress, in which the searchlight of scientific medicine is casting its rays into the hidden recesses of medical science, and shedding light on many an abstruse problem, which sorely perplexed the scientist of the past, but we should feel justly proud and honored that we are members of the noblest, the most humane, the most beneficent, and the most progressive of all the professions; and as we speed past the mile-post of medical progress we must not loiter lazily in the alluring shades of idleness, nor rest contentedly on flowery beds of ease. Neither should we linger long in the whirlpool of pleasure, lest we become desuetude and unserviceable, and thereby deserve to be Oslerized; for knowledge is now the standard of worth, and reward clings closely to merit.

